# Maternal or Paternal Diabetes and Its Crucial Role in Offspring Birth Weight and MODY Diagnosis

**DOI:** 10.3390/metabo10100387

**Published:** 2020-09-28

**Authors:** Valeria Calcaterra, Angela Zanfardino, Gian Vincenzo Zuccotti, Dario Iafusco

**Affiliations:** 1 Pediatric and Adolescence Unit, Department of Internal Medicine and Therapeutics, University of Pavia, 27100 Pavia, Italy; 2 Pediatric Unit, “V. Buzzi” Children’s Hospital, 20154 Milano, Italy; gianvincenzo.zuccotti@unimi.it; 3 Department of Pediatrics, Regional Center of Pediatric Diabetology “G. Stoppoloni”, University of Campania “Luigi Vanvitelli”, 80138 Napoli, Italy; angela.zanfardino@unicampania.it (A.Z.); dario.iafusco@unicampania.it (D.I.); 4 Department of Biomedical and Clinical Sciences “L. Sacco”, University of Milan, 20157 Milano, Italy

**Keywords:** mother, father, diabetes, birthweight, MODY

## Abstract

Maturity-onset diabetes of the young (MODY) represents a heterogenous group of monogenic autosomal dominant diseases, which accounts for 1–2% of all diabetes cases. Pregnancy represents a crucial time to diagnose MODY forms due to the 50% risk of inheritance in offspring of affected subjects and the potential implications on adequate fetal weight. Not only a history of maternal diabetes may affect the birth weight of offspring, paternal diabetes should also be taken into consideration for a correct pathogenetic diagnosis. The crucial role of maternal and paternal diabetes inheritance patterns and the impact of this inherited mutation on birthweight and the MODY diagnosis was discussed.

Maturity-onset diabetes of the young (MODY) represents a heterogenous group of monogenic autosomal dominant diseases, which accounts for 1–2% of all diabetes cases [1]. However, an exhaustive search of the literature on the topic of diabetes pathogenesis in select populations has shown that the actual number of cases should be much higher. In an Italian pediatric case series, studied in highly qualified centers, up to 6.3% of patients were diagnosed with monogenic diabetes [2]. More than a dozen MODY genes have been identified to date. All of these genes are associated with impaired insulin secretion and are of great importance in the diagnosis and molecular classification of MODY as well as treatment decisions [3,4].

Even if most MODY cases are characterized by a reduction in insulin production and the classic phenotype is generally observed in underweight people, we have recently demonstrated that the phenotype may be strongly influenced by the environment and may also be common in obesity [2].

Pregnancy represents a crucial time to diagnose MODY forms due to the 50% risk of inheritance in offspring of affected subjects and the potential implications on adequate fetal weight [5,6,7]. 

Not only a history of maternal diabetes may affect the birth weight of offspring, paternal diabetes should also be taken into consideration for a correct pathogenetic diagnosis.

A maternal anamnestic history including: pregestational hyperglycemia, maternal weight before gestation and/or absence of excessive gestational weight gain, absence of signs or symptoms suggestive of different types of diabetes, one parent affected by diabetes of any type or impaired fasting glucose with or without impaired glucose tolerance, abnormalities of the placenta and baby birth weight all represent important factors for an accurate diagnosis [8,9].

Insulin secretion is a major determinant of intrauterine growth [10]. About 15–45% of babies born to diabetic mothers may have macrosomia, which is a 3-fold higher glycemic rate compared to normoglycemic controls. According to the modified Pedersen’s hypothesis, when maternal glycemic control is impaired and the maternal serum glucose level is high, glucose crosses the placenta [11]. The hyperglycemia induced in the fetus increases the production of fetal insulin while maternal insulinemia has no effect, as this hormone does not cross the placental barrier [12].

In the second trimester, the fetal pancreas responds to hyperglycemia and secretes insulin in an autonomous manner (hyperinsulinemia). The combination of hyperinsulinemia and hyperglycemia in the child of a diabetic mother leads to increases in protein and fat stores in the fetus, resulting in macrosomia. However, this condition is not obvious in the fetuses of pregnant MODY women and several outcomes (scenarios) are possible.

## 1. MODY-2 Scenario 

### Glucokinase GCK Mutation 

GCK acts as a pancreatic glucose sensor [2,7]. Heterozygous inactivating *GCK* mutations, otherwise known as MODY-2, manifest in mild fasting hyperglycemia from birth. Since fasting hyperglycemia is usually asymptomatic, pregnancy may often be the first occasion to detect it.

In a patient with MODY-2 (GCK deficit), low or high birth weights may result. Normal GCK function is essential for normal fetal insulin secretion. Non-affected fetuses of GCK-deficient mothers as well as any child from a mother with diabetes may present with a high birth weight (large gestational age—LGA).

When, on the other hand, the fetus inherits the maternal mutation, and this happens in 50% of cases, it will have a higher glucose sensor setpoint, so it will need higher maternal glycemia to stimulate normal insulin secretion to achieve a normal birth weight. Therefore, hypoglycemic therapy in MODY 2 pregnant women is not recommended if the fetus is also affected. Homozygous inactivating *GCK* mutations have also been reported to cause severe hyperglycemia, presenting as permanent neonatal diabetes mellitus with low birth weights in the newborn.

## 2. MODY-1 Scenario 

HNF4A Mutations

Changes in the *HNF4α* gene (MODY-1) cause the production of a high number of pancreatic beta-cells with hyperinsulinemia before birth, and the newborn is usually LGA [3]. The affected infants exhibit macrosomia and transient neonatal hypoglycemia due to hyperinsulinism during fetal and neonatal life, with a switch to defective insulin secretion later in life due to apoptosis of beta cells [3,7]. In the case of a non affected fetus of a mother carrying a mutation, LGA may occur in the absence of adequate treatment similarly to MODY-3. Finally, with the birth of a LGA newborn from a father with diabetes and a normal mother, MODY-1 suspicion is well founded.

## 3. MODY-3 Scenario 

### HNF1A Mutations

Heterozygous *HNF1A* mutations cause pancreatic-islet β-cell dysfunction and monogenic diabetes (MODY-3) [3]. In the non affected fetus of mothers carrying an HNF-1α mutation, near-normalization of maternal glycemic control during pregnancy is recommended to prevent LGA.

In fetuses carrying an HNF-1α mutation or paternal inherited mutation, normal birth weights have been described, but exposure to intrauterine hyperglycemia can lead to early diabetes onset [3,7]. In fact, MODY-3 patients who inherit the mutation from their father and experience a gestational euglycemic environment before birth develop diabetes later than patients who inherit the disease from their mother (gestational hyperglycemic pregnancy) [8,9].

Therefore, a MODY-3 fetus requires near-normalization of maternal glycemic control. Neonatal hypoglycemia is generally observed in MODY-1 infants, but it is possible to hypothesize that some HNF-1α mutations could lead to a functionally impaired protein that might dysregulate HNF-4α expression determining hypoglycemia.

Recently we described dizygotic twins discordant for the mutation from a mother with MODY-3. The twin with the HNF1alpha mutation had a higher birth weight and suffered from hypoglycaemia soon after birth.

## 4. MODY-5 Scenario 

### HNF1β Changes (Mutations/Deletions)

Renal cysts and diabetes syndrome (MODY-5) are usually noted in patients with the HNF1β mutation [13]. In this case, diabetes develops during adolescence or early adulthood and usually progresses to an insulin-dependent state due to pancreatic hypoplasia, with hepatic insulin resistance, in a relatively earlier period of the disease. The carriers of HNF1β mutations have significantly reduced birthweights [3]. The normal birthweights observed in non-affected fetuses of mothers carrying the mutation are related to glycemic control in the mother.

These scenarios show the crucial role of maternal and paternal diabetes inheritance patterns and the impact of this inherited mutation on birthweight and the MODY diagnosis. In the presence of maternal diabetes during pregnancy, it is mandatory to consider a MODY diagnosis in order to define the appropriate therapeutic approach to prevent both LGA and SGA. Additionally, anamnestic data regarding the diabetes history of the father should always be considered. 
